# Transcriptome Analysis of Wounding in the Model Grass *Lolium temulentum*

**DOI:** 10.3390/plants9060780

**Published:** 2020-06-22

**Authors:** James E. Dombrowski, Brent A. Kronmiller, Vicky Hollenbeck, Ruth C. Martin

**Affiliations:** 1 USDA-ARS, National Forage Seed Production Research Center, 3450 SW Campus Way, Corvallis, OR 97331-7102, USA; dombrowskijim2020@gmail.com (J.E.D.); Vicky.Hollenbeck@usda.gov (V.H.); 2 Center for Genome Research and Biocomputing, Oregon State University, Corvallis, OR 97331, USA; Brent.Kronmiller@cgrb.oregonstate.edu

**Keywords:** brassinosteroid, grass, JA, *Lolium*, MAPK, pathogen, receptor, signaling, transcriptome, wounding

## Abstract

For forage and turf grasses, wounding is a predominant stress that often results in extensive loss of vegetative tissues followed by rapid regrowth. Currently, little is known concerning the perception, signaling, or molecular responses associated with wound stress in forage- and turf-related grasses. A transcriptome analysis of *Lolium temulentum* plants subjected to severe wounding revealed 9413 upregulated and 7704 downregulated, distinct, differentially expressed genes (DEGs). Categories related to signaling, transcription, and response to stimuli were enriched in the upregulated DEGs. Specifically, sequences annotated as enzymes involved in hormone biosynthesis/action and cell wall modifications, mitogen-activated protein kinases, WRKY transcription factors, proteinase inhibitors, and pathogen defense-related DEGs were identified. Surprisingly, DEGs related to heat shock and chaperones were more prevalent in the downregulated DEGs when compared with the upregulated DEGs. This wound transcriptome analysis is the first step in identifying the molecular components and pathways used by grasses in response to wounding. The information gained from the analysis will provide a valuable molecular resource that will be used to develop approaches that can improve the recovery, regrowth, and long-term fitness of forage and turf grasses before/after cutting or grazing.

## 1. Introduction

Grasses are subjected to multiple biotic and abiotic stresses throughout their life cycle. They must be able to sense the different types of stress and respond appropriately to alter cellular, metabolic, and physiological processes to adapt and survive these stresses. Plants have evolved a variety of interconnected networks and mechanisms for perceiving, signaling, and responding to these various stresses. For forage and turf grasses, wound stress is one of the most predominant stresses that they are exposed to on a continual basis. Damage or loss of plant tissue occurs when plants are crushed by treading, grazing, feeding insects, or cutting. Most of these wound stresses result in extensive loss of vegetative tissues and rapid regrowth. The molecular characterization of the wound stress response has been most extensively carried out in dicotyledonous plant systems [1,2,3,4]; however, it has not been as well characterized in monocots.

Plants respond to wounding by generating a diverse array of signals, which in turn activate complex, integrated signaling networks [2,5,6,7,8,9,10,11,12,13,14,15,16]. A plant perceives wound damage through plant-derived compounds and signals generated at the wound site or from elicitors found in the secretions from herbivores. These signals lead to changes in gene expression patterns and the synthesis of proteins and compounds locally and systemically that alter the plant’s physiological and metabolic state [1,2,4,17]. Wound-generated signals are transmitted systemically to distal portions of the plant by hydraulic, electrical, and/or chemical signals [7,8,9,10,12,14]. In addition to proteins such as kinases, receptors, calmodulin, calcium-binding proteins, and transcription factors for signaling, small molecules such as reactive oxygen species (ROS), calcium, ethylene, salicylic acid (SA), and jasmonic acid (JA) derivatives are also integral in the wound response [2,5,6,7,8,9,10,11,12,13,14,15,16]. JA and its bioactive derivatives play an essential role in wound signaling [7,15,18,19,20]. JA has also been shown to play a significant role in many aspects of growth, development, and environmental responses in plants [7,20,21]. Additionally, there are JA-independent signaling pathways that modulate the expression of JA-responsive genes and regulate the expression of distinct sets of wound-related genes [7,16,22,23]. Volatile compounds are another important class of wound signaling molecules [24,25,26,27,28,29,30,31,32]. These plant volatiles may prime or enhance plants’ wound response via inter- and/or intra-plant signaling [30,31,33,34,35,36,37,38,39,40,41,42]. When grasses are mechanically damaged, they release a volatile chemical blend called green leaf volatiles (GLVs) into the surrounding atmosphere [32,43]. GLVs are composed of six-carbon compounds that include esters, aldehydes, and alcohols, the composition of which has been described for tufted hairgrass [32].

While the wound response in monocots is not well characterized, there is evidence that monocots and dicots utilize many of the same signaling components. Some examples of similar signaling elements found in dicots and cereal grasses include: various components of oxylipin biosynthesis and their role in wounding in maize and rice [44,45,46,47,48]; electrical and hydraulic signals identified in barley [49,50]; mitogen-activated protein kinases (MAPKs) in rice [51,52]; volatile organic compounds released in various monocot species [30,32,53,54]; wound-inducible genes and proteins in maize and rice [30,55,56,57,58,59,60]; and defense-related proteins such as proteinase inhibitors in maize and *Brachypodium* [61,62,63].

Molecular mechanisms associated with wound responses in forage and turf grasses are starting to be revealed. Le Deunff et al. [64] showed an oxidative burst in leaf blades of ryegrass after wounding. In sheepgrass, a comparative analysis of transcriptomes generated from mechanical wounding and defoliation identified a wide range of genes and metabolic pathways affected by these stresses [65]. Mechanical wounding in six different forage and turf grass species rapidly activated both a 46 kDa MAPK and a 44 kDa MAPK; and in *Lolium temulentum* (*Lt)*, wounding activated the 46 kDa MAPK locally and systemically in an adjacent tiller within five minutes [66]. Furthermore, activation of these MAPKs occurred with exposure of undamaged plants to GLVs released from cut leaf blades; only one minute of exposure activated the *Lt* 46 kDa MAPK and the *Lt* 44 kDa MAPK within three minutes and fifteen minutes post-exposure, respectively [67,68]. Additionally, the *Lt* MAPKs were activated by exposure to just a single compound from a variety of chemical classes contained within the GLV mixture, as well as from exposure to GLVs released from an unrelated plant species [68]. These results suggest that the MAPKs are activated as part of a general response to the release of GLVs from damaged plants in their environment. Recently, an analysis of the *Lt* GLV transcriptome revealed a rapid, but transient, induction of over 4000 genes, with strong emphasis on signaling-related genes, within two hours of exposure. Furthermore, an analysis of selected genes found to be upregulated in the GLV transcriptome showed strong induction by mechanical wounding. The analysis of the *Lt* GLV transcriptome combined with previous studies suggests that GLVs released from cut grasses not only affect the injured plants, but can also transiently prime wound stress pathways in nearby undamaged plants, possibly alerting them to potential oncoming damage [42]. While the analysis of the GLV transcriptome gives insight into the perception, potential signaling pathways, and networks used to respond to wound stress, it lacks information on the processes that the plants utilize to effect repair and regrowth of lost tissue, and the type of defense-related proteins and compounds they produce.

In this study, we used the model grass *Lt,* a diploid self-pollinating species that is closely related to forage and turf grass species, many of which are obligate outcrossing species, to reduce the inherent variability present between individuals in those heterogenous populations. We investigated the transcriptional profile of the model grass *Lt*, subjected to severe wounding and extensive loss of vegetative tissue. We generated an RNA-Seq transcriptome to identify genes, as well as metabolic and signaling pathways, that were induced. The analysis of the wound-induced *Lt* transcriptome revealed a wide range of sequences coding for proteins involved in signaling, growth, and stress-related pathways. This research provides new information on the molecular components and pathways used by forage- and turf-related grasses in response to wounding.

## 2. Results and Discussion

### 2.1. RNA-Seq Libraries

The RNA-Seq *Lt* wound transcriptome was generated from severely wounded plants with a significant loss of vegetative tissue. Sequencing produced from 37.3 M to 74.6 M reads for control plants and from 47.1 to 83.1 M reads for wounded plants per sample. The initial read counts and percent alignments for expression libraries generated from control and wounded *Lt* plants are summarized in Table 1. The *Lolium* transcriptome previously used to assemble the GLV transcriptome [42] was used as a reference transcriptome to assemble the wound transcriptome and is shown in Table S1. The results of wounding were evaluated by comparing the values derived for the differential expression of sequences (fragments per kilobase million (FPKM)) between the wounded plants and their corresponding untreated controls at the 1, 2, 6, 12, and 24 h time points. As with the GLV transcriptome [42], the false discovery rate (FDR) was set at ≤0.05 and the *p*-value was ≤0.01; and we converted “0” values for FPKM to 0.05, where appropriate. The results of the analyses were separated into upregulated and downregulated datasets.

**Figure 1:**
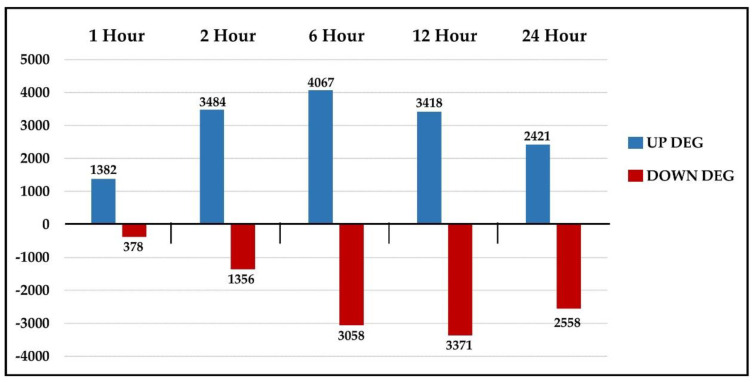
Total differentially expressed genes (DEGs) per time point. Graph depicting the total number of upregulated and downregulated DEGs identified at each time point. Abbreviations: UP DEG, upregulated differentially expressed genes; DOWN DEG, downregulated differentially expressed genes.

Using the cut-offs described above, we identified 14,772 upregulated and 10,721 downregulated DEGs in the combined (1, 2, 6, 12, and 24 h) time-point dataset, with 9413 unique upregulated DEGs and 7704 unique downregulated DEGs. The use of existing databases for other plant species resulted in the annotation of 77.1% of the total DEGs identified. As shown in Figure 1, the analysis of the total DEGs over the course of the study showed 1382 upregulated DEGs and only 378 downregulated DEGs after the first hour post-wounding compared to the control. After two hours post-wounding, there was a 2.5-fold increase in the upregulated DEGs (3484) and a 3.59-fold increase in the downregulated DEGs (1356) compared to the first hour. Despite this substantial increase in the number of downregulated DEGs from the 1 h to 2 h time point, these downregulated DEGs only represented 28% of the total DEGs identified at the 2 h time point. The upregulated DEGs reached their maximum level after 6 h (4067) and slowly decreased by 40% over the next 18 h, to 2421 at the 24 h time point. In contrast, the downregulated DEGs increased substantially (2.49-fold) over the next 4 h, reaching their maximum at the 12 h time point (3371), and then decreased by 24% to 2558 downregulated DEGs at the 24 h time point. The identified nucleotide sequences, sequence lengths, annotations, fragments per kilobase million (FPKM), log_2_ fold changes (up and down) and *p*- and *q*-values for wound-treated samples and their corresponding untreated controls for each time point are listed in Table S2.

### 2.2. Gene Ontology Enrichment Analyses

**Figure 2:**
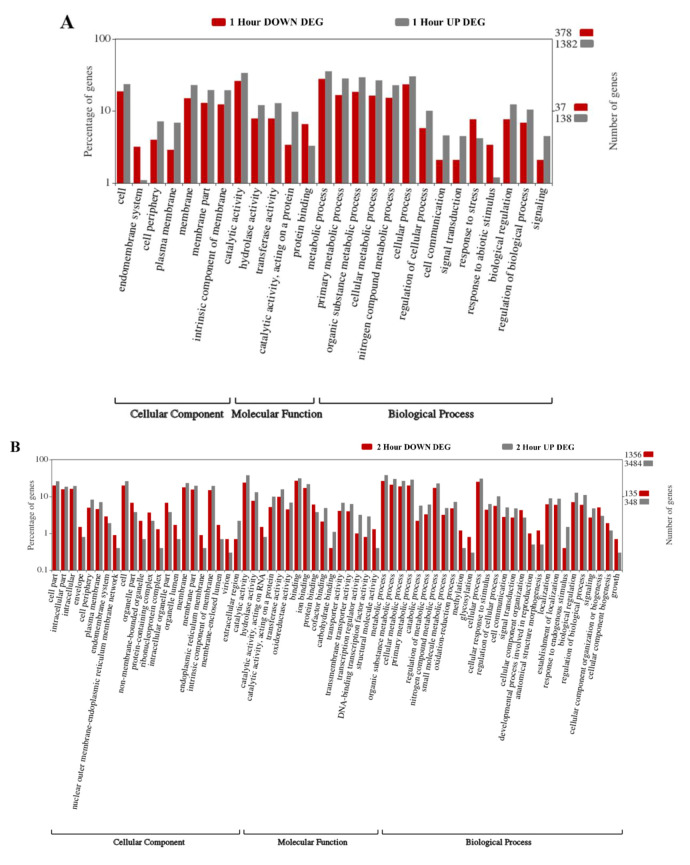

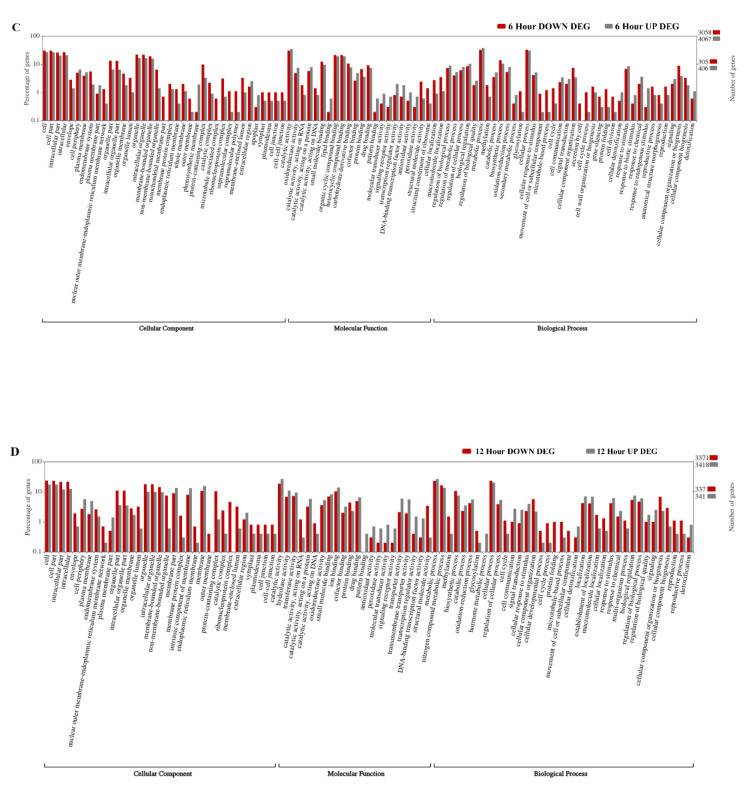

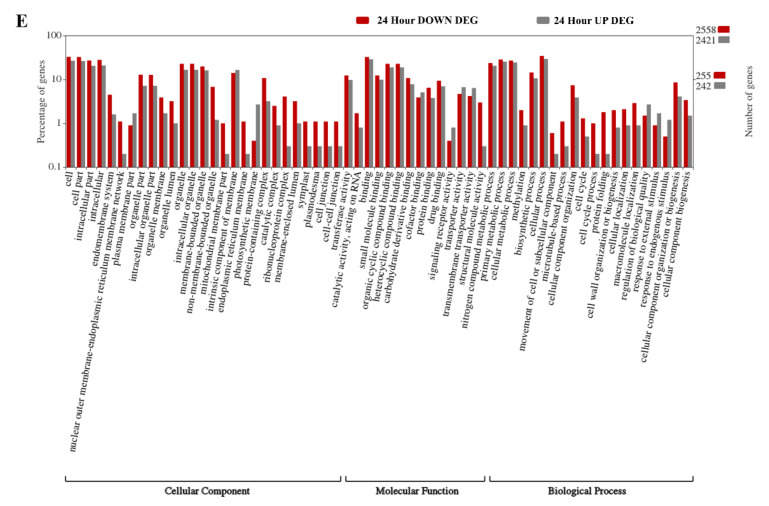
Gene ontology analysis for upregulated and downregulated DEGs of wound databases for (**A**) 1 h post-wound, (**B**) 2 h post-wound, (**C**) 6 h post-wound, (**D**) 12 h post-wound, and (**E**) 24 h post-wound. Red bars = percentage of downregulated DEGs, gray bars = percentage of upregulated DEGs. Numbers on the right-hand axis represent the percentage of genes in log_10_ scale. Numbers on the left axis are the total number of DEGs contained in upregulated (gray) and downregulated (red) datasets used in the analysis. Abbreviations: UP DEG, upregulated differentially expressed genes; DOWN DEG, downregulated differentially expressed genes.

A gene ontology (GO) enrichment analysis was used to derive functional information from the DEG datasets. Figure 2 shows GO classifications of the DEGs for 1, 2, 6, 12, and 24 h post-wound time points, separated into three categories: cellular component, molecular function, and biological process. Not surprisingly, the number of subcategories per time point was also related to the number of DEGs being analyzed, with the 6 and 12 h categories (Figure 2C,D) having a greater number of categories represented than the 1, 2, and 24 h categories (Figure 2A,B,E). As we evaluated the GO term analysis, we focused our attention on those subcategories that had the highest levels of DEGs associated with them and those categories displaying the largest differential between upregulated and downregulated DEGs as potential discriminators. The GO subcategories that were found to have the highest levels of DEGs throughout the study were those related to cell and metabolic processes. In addition to those present in all categories, we found categories associated with binding and organelles to be highly represented in all time points after one hour. Most of the GO subcategories one hour after wounding appeared to be enriched for upregulated DEGs, except for protein binding, endomembrane system, and surprisingly, response to stress and abiotic stimulus. After two hours (Figure 2B), in addition to those subcategories previously mentioned, membrane, catalytic activity, and cellular process were enriched overall. While most of the subcategories displayed higher levels for upregulated DEGs, the most enriched subcategories for upregulated when compared to downregulated DEGs were extracellular regions, transcription-related categories, and response to endogenous stimulus. A higher level of downregulated DEGs was displayed by 22 of the 66 subcategories after two hours, the most prominent being non-membrane bounded organelle, ribonucleoprotein complex, structural molecular activity, methylation, glycosylation, developmental process involved in reproduction, structural morphogenesis, and growth. After six hours post-wounding, the GO term analysis expanded to 99 subcategories (Figure 2C), and increases in total DEGs in catalytic activity, biosynthetic activity, and response to stimulus were observed. We observed increases in upregulated DEGs in categories related to signaling, transcription, response to stimulus, and detoxification (peroxidase and antioxidant activity) and in DEGs involved in photosynthetic membrane. Most other membrane-related categories, such as nuclear-endoplasmic reticulum (ER) membrane network, mitochondrial membrane part and outer membrane, as well as microtubule, supramolecular and ribonucleoprotein complexes, methylation, glycosylation, protein folding, and cell cycle displayed enrichment for downregulated DEGs. At twelve hours, the number of subcategories remained high at 92 (Figure 2D), with a number of membrane subcategories displaying a high level of DEGs. Categories related to transporters, transcription, signaling, and detoxification showed enrichment for upregulated DEGs; and methylation, nuclear-ER membrane network, catalytic activities on RNA and DNA, protein folding, cycle process, and reproduction showed higher enrichment for downregulated DEGs.

At the 24 h time point, the subcategories decreased to 61, with only nine subcategories showing higher levels of upregulated DEGs to downregulated DEGs (Figure 2E). DEGs involved in photosynthetic membrane, transporter categories, signaling receptor activity, and response to stimulus were enriched for upregulated DEGs; and non-membrane organelle, mitochondrial membrane part, structural molecule activity, nuclear-ER membrane network, ribonucleoprotein complexes, symplast, plasmodesma, cell-to-cell junctions, and protein folding showed the highest enrichment for downregulated DEGs.

### 2.3. Comparative Analysis of DEGs at Different Time Points

**Figure 3:**
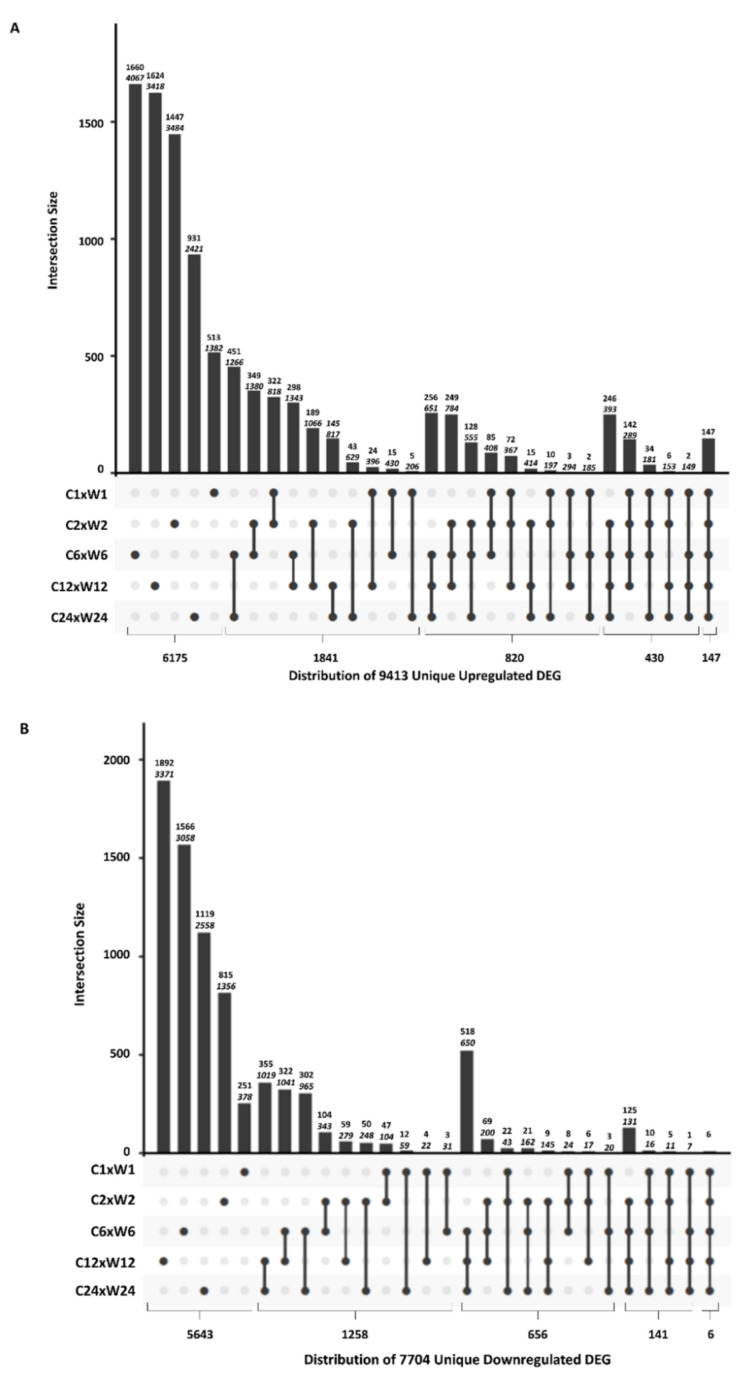
UpSetR plots depicting the number of unique and shared differentially expressed genes (DEGs) between control (C) and wounded (W) samples for each time point. Graph depicts the unique, total, and shared number of (**A**) upregulated DEGs and (**B**) downregulated DEGs for each time point. The top number above each bar represents the number of DEGs that are unique to each group. The bottom italicized number above the bar represents the total number of DEGs in that category, including those shared with other time points. Bars represent the number of DEGs shared between post-wound time points indicated by linked dots below the x-axis. The numbers below the chart represent the total number of DEGs present in only one, two, three, four, or all five time points.

In order to investigate the shared upregulated or downregulated DEGs between time points, comparisons of DEGs between time points were performed. As shown in Figure 3A (upregulated DEGs) and Figure 3B (downregulated DEGs), there were significant overlaps of shared DEGs present between the different time points. The largest number of shared upregulated DEGs (1380) for consecutive time points was between the 2 and 6 h time points, representing approximately 36% of the DEGs found in the 2 h time point. In the downregulated DEGs, approximately 30% (1019) of the DEGs in the 12 h dataset were also found in the 24 h dataset. Only 817 upregulated DEGs were shared between 12 and 24 h time points, with approximately 24% of the 12 h upregulated DEGs being shared with the 24 h time point. For the downregulated DEGs, 104 DEGs were shared between the 1 and 2 h time points, and approximately 28% of the downregulated DEGs from the 1 h time point were shared with the 2 h time point.

Additionally, we investigated how prevalent a specific DEG was over the course of the study (Figure 3A,B). Of the 14,772 combined upregulated DEGs and 10,721 combined downregulated DEGs, 9413 (63.7% of the upregulated DEGs) and 7704 (71.9% of the downregulated DEGs) were unique sequences. Furthermore, 65.6% (6175) of upregulated DEGs and 73.2% (5643) of downregulated DEGs were represented only once in the combined datasets (Figure 3A,B; Table S3).

Only 1.6% (147) of upregulated DEGs and only 0.08% (6) of downregulated DEGs were present in every time point (Figure 3A,B; Table S3). It was surprising to find such a large percentage of DEGs to be present in only a single time point. It should be noted that some of the unique DEGs may also occur in other time points, but were potentially excluded from the datasets because one or more of the parameters were below the cut-offs used to create the dataset.

**Figure 4:**
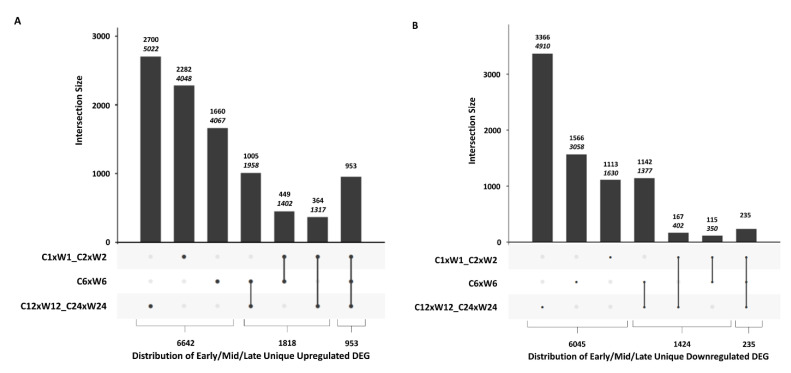
UpSetR plots depicting the number of unique and shared differentially expressed genes (DEGs) between control (C) and wounded (W) samples for early (1 h plus 2 h time points), mid (6 h time point), and late (12 h plus 24 h time points) response periods. Graph depicts the unique, total, and shared number of (**A**) upregulated DEGs and (**B**) downregulated DEGs for each response period. The upper number above each bar represents the number of DEGs that are unique to each group. The lower italicized number above the bar represents the total number of DEGs in that category, including those shared with other time points. Bars represent the number of DEGs shared between post-wound time points indicated by linked dots below the x-axis. The numbers below the chart represent the total number of DEGs present in only one, two, or all three time periods.

To gain insight into the overlap of DEGs between early, mid, and late DEGs, an UpSetR plot was generated (Figure 4A,B). Early DEGs were represented by combining the 1 and 2 h datasets, mid DEGs by the 6 h dataset, and late DEGs by combining the 12 and 24 h datasets. Of the 9413 unique upregulated DEGs, 10.1% (953) were found to be upregulated in all three datasets, while 28.7% (2700) were found only in the late dataset, 24.2% (2282) only in the early dataset, and 17.6% (1660) only in the mid dataset (Figure 4A; Table S4). Of the 7704 unique downregulated DEGs, only 3.1% (235) were found in all three datasets, while 43.7% (3366) were found only in the late dataset, with 20.3% (1566) found only in the mid dataset, and only 14.4% (1113) were unique to the early time point (Figure 4B; Table S4). In both cases, the mid and late datasets shared the most upregulated and downregulated DEGs. Further investigation into the DEGs of the late datasets found that over 50% of the upregulated and 42.2% of the downregulated DEGs found exclusively in the late datasets had no annotation. It would be interesting to determine the role these DEGs play in the wound response. Data for the upregulated and downregulated DEGs corresponding to the early-mid-late Venn analyses and their values are available in Table S4.

In order to determine if any of the DEGs were both upregulated and downregulated over the course of the study, a Venn analysis was performed on the combined upregulated and downregulated datasets. Only 239 DEGs or 2.5% of the upregulated DEGs were also found in the downregulated dataset. The upregulated and downregulated DEGs corresponding to the upregulated vs downregulated DEG Venn analyses and their values are listed in Table S4.

### 2.4. DEG Categories

The GO analysis revealed an emphasis in the upregulated datasets for transporter activity, signaling, transcription, detoxification, and photosynthesis subcategories as compared to the downregulated dataset. The emphasis of downregulated DEGs in comparison with the upregulated dataset was in areas associated with protein folding, ER membrane network and reproduction subcategories. In order to gain further insights, we conducted keyword searches (shown in Table 2) of the combined upregulated and downregulated DEG datasets to further identify potential components associated with signaling, biosynthetic, and metabolic pathways impacted by wounding. Most of the functional categories listed in Table 2 displayed significantly more upregulated DEGs per category than downregulated DEGs, except for heat shock/chaperone, ferric reductase, expansin, and GTPase. This was also true when looking at log_2_ fold differences greater than 2 and less than –2, as shown in Table 2. Signaling is a crucial component to a plant’s response to stress; a plant utilizes a variety of signaling molecules to mediate its response to wounding. JA and its derivatives are key signaling molecules regulating the plant’s responses to wounding and a wide range of stresses, growth, and development [7,19,21,22,69,70]. As shown in Table 2, genes encoding many of the enzymes in the JA biosynthetic pathway [20,71,72,73], including phospholipase (34 up (13 phospholipase A), 17 down), lipoxygenases (24 up, 12 down), allene oxide synthase (6 up, 0 down), allene oxide cyclase (1 up, 1 down), and 12-oxophytodienoic acid reductase (8 up, 1 down), were predominantly found in the upregulated DEG dataset as compared to the downregulated dataset. Many of these same genes were also upregulated in response to GLVs [42], and a similar upregulation of JA biosynthesis genes has been reported in *Arabidopsis* in response to wounding [4]. Furthermore, methyl jasmonate has been shown to induce defense responses in gymnosperms via terpenoid biosynthesis, the development of traumatic resin ducts, and polyphenolic parenchyma cells [74,75,76,77,78,79,80,81]. The JA biosynthetic pathway was also induced in response to wounding and feeding by spruce budworm or white pine weevils in Sitka spruce [82]. Interestingly, JA biosynthetic genes were not found to be differentially regulated during wound xylem formation in *Pinus canariensis* [83], but this may be due to the later sampling time (7 days after wounding). Clearly, JA is an important component of the wound response across a wide range of plant species.

Another hormone involved in wound and pathogen responses is ethylene. Twenty-five upregulated DEGs coding for a key enzyme for ethylene biosynthesis, 1-aminocyclopropane-1-carboxylate (ACC) oxidase [84], were found in the wound database. The number of upregulated ACC oxidase DEGs increased over time with a dip at 12 h (5, 9, 12, 8, and 13 DEGs at 1, 2, 6, 12, and 24 h post-wounding, respectively) (Table 3). Compared to a recent study analyzing the effect of green leaf volatiles (GLVs), there were fewer DEGs for ACC oxidase genes present in the GLV upregulated genes and they were present at the 1 and 2 h post-exposure time points [42]. Ethylene biosynthetic genes are also upregulated in conifer species in response to mechanical wounding (24 h) and weevil chewing (48 h) [82]. Interestingly, the expression of ethylene biosynthetic genes is also seen seven days after wounding, the time frame when the first traumatic wound tissue is thought to be formed, and at the later stages of wound xylem formation [83]. The upregulation of ethylene biosynthesis at early time points in response to GLVs, wounding, and insect damage support the role of ethylene as an early signaling molecule in the wound response, but the sustained presence at later stages may suggest a further role in the healing and growth process.

Genes coding for calcium- and calmodulin-interacting proteins, which are also essential signaling components in a plant’s response to wounding and other stresses [8,85,86], were also found to be upregulated in response to wounding in *Lt*. These upregulated DEGs encoded mainly calcium/calmodulin-binding proteins, calcium-dependent protein kinases, and calcium-transporting ATPases. The peak level for total and for each group of calcium-interacting proteins was found at the 2 h post-wound time point and 1 h after exposure to GLVs [42]. Calcium plays a role in the production of ROS [87], which are produced in response to wounding and play a key role in stress signaling [8,14,88,89,90,91], but can also lead to cell death if present in excess. Several DEGs coding for NADPH oxidases (respiratory burst oxidase homolog D (RBOHD)), which play a key role in ROS production [14], were found to be upregulated at one and two hours after wounding (Table 3).

Peroxidases, which are also involved in the generation of a wound-induced burst of ROS [92], were represented by 57 DEGs in the upregulated and 32 DEGs in the downregulated wound datasets. Over half of the upregulated peroxidase DEGs were found at the 6 h post-wounding time point (Table 3); and previously, peroxidases were found to be upregulated after just 1 h of exposure to GLVs [42]. Interestingly, DEGs of glutathione S-transferases, which are involved in detoxifying xenobiotics and protecting the cell from oxidative damage, were also upregulated mainly at the 2 and 6 h post-wound time points (Table 3) and after 1 h of exposure to GLVs [42]. Thioredoxin is another protein involved in ROS scavenging, and thioredoxin-encoding DEGs were found to be upregulated at the early time points, increasing from the 1 h (2/3; 2 with log_2_ fold > 2, out of 3 total DEGs) to 2 h (4/5) and 6 h (5/17) post-wounding time points and then decreasing gradually (Table 3), while in an earlier study, thioredoxin DEGs were only present in the 1 h GLV dataset [42]. The presence of ROS production and ROS scavenging related DEGs is probably essential to maintain signaling, but to minimize potential damage due to excess ROS signaling molecules.

Protein kinases play an essential role in a plant’s responses to stress and in the regulation and activation of a wide variety of cellular processes within the cell [93,94]. Our analysis of the datasets revealed that 637 (6.8%) of the upregulated DEGs and 348 (4.5%) of the downregulated DEGs were annotated as kinases, which are integral proteins for transmission of signals within the cell and would be expected to be predominant early in the response to a stress. An analysis of the distribution of kinase DEGs revealed that the highest number of upregulated kinases were present at 2 h (~50%, 321 DEGs) and 6 h (~43%, 272 DEGs) after wounding (Table 3). Furthermore, phosphatases, which work antagonistically with kinases in regulating a wide range of processes and functions within the cell, were annotated to 169 DEGs in the upregulated and 76 DEGs in the downregulated datasets. As shown in Table 3, the distribution of the upregulated phosphatase DEGs at the different time points mirrored that of the kinases, with their highest levels also present at the 2 h (82 DEGs) and 6 h (83 DEGs) time points.

The functional role of mitogen-activated protein kinases (MAPKs) in the wound response has been widely described in various plant species [52,95,96,97]. Previously, we found that *Lt* MAPKs are rapidly activated in plants exposed to wounding, GLVs, and a variety of other abiotic stresses [66,67,68,98]. A 46 kDa MAPK was activated locally and systemically in an unwounded tiller within 5 min of wounding in the model grass *Lt* [66]. Furthermore, just a one-minute exposure to GLVs released from cut grass leaf blades was enough to activate the *Lt* 46 kDa MAPK in adjacent undamaged *Lt* plants within 3 min and the 44 kDa MAPK within 15 min [67,68]. Additionally, thirteen different commercially available plant volatile compounds, as well as GLVs derived from damaged leaf tissues of three other grass species and tomato, were shown to activate these MAPK in *Lt* [68]. In our previous transcriptome analysis of plants exposed to GLV, we found that 50% of the GLV-upregulated MAPK DEGs were MAPKKK (5 MAPK, 5 MAPKK, and 10 MAPKKK) [42]. Our current analysis identified 18 MAPK DEGs (12 MAPK, 3 MAPKK, and 3 MAPKKK) in the upregulated and 6 MAPK DEGs in the downregulated wound datasets. A comparison of the wound- and GLV-induced MAPK DEGs found that only six of the upregulated MAPKs (5 MAPK and 1 MAPKK) were common under both stresses. They may be involved in perception and early signal transmission initiating the wound response, while the other MAPK DEGs identified in the wound transcriptome may have a role in the regulation of other wound-related processes. For instance, some MAPKs are involved in regulating various aspects of plant growth [99,100,101]. In *Arabidopsis thaliana* root cells, a MAPK (MPK4) was found to be co-localized with microtubule arrays, and was shown to play a role in the transition from mitosis to cytokinesis [102]. Furthermore, a MAPKKK (MEKK1) was shown to mediate reactive oxygen species homeostasis in *Arabidopsis*, with the MPK4 as its downstream target [103]. Therefore, some of the MAPK DEGs identified in the transcriptome may not be involved in the early signaling events, but may be involved in regulating other aspects of the plants’ response to wounding, for example, cellular growth.

Receptor proteins are key signaling components that sense changes in the environment or respond to a wide range of molecules associated with stress or development, leading to a specific response, which results in alterations in the physiological and metabolic state of the cell and plant. An analysis of the transcriptome revealed 431 receptor DEGs in the upregulated dataset, of which 281 (65%) were annotated as receptor kinases; and 236 receptor DEGs in the downregulated dataset, with 112 designated as receptor kinases. Further analysis of the upregulated DEG database revealed eight receptor DEGs designated as SR160, a leucine-rich repeat receptor kinase (LRR-RK). This LRR-RK was originally believed to be the systemin receptor [104], but actually is a homolog of the tomato brassinosteroid insensitive 1 receptor (BRI1) [105]. Brassinosteroid hormones are critical for plant growth and development and are involved in controlling cellular division, elongation, and differentiation [106,107]. Furthermore, these hormones have been shown to play an important role in plant adaptation to environmental stress [106,107]. There were eight additional wound-upregulated and three wound-downregulated DEGs in the brassinosteroid family of receptors, suggesting that they may have a significant role in recovery and growth after wounding in grasses. In *Arabidopsis* roots, a small group of stem cells located at the base of the meristem in the root apex is essential for sustaining root growth. These precursors are used for growth or as a source of cells to replace tissues that have been damaged [reviewed in 106]. Brassinosteroids are involved in the regulation of the dormancy and differentiation of these precursor root stem cells. In grasses, the root crown is the center for sustaining the growth of leaf tissues, with the meristematic region being located at the base of the pseudostems. Cut grasses respond to the loss of tissue with rapid growth to replenish lost vegetative tissue. Potentially, brassinosteroids could provide a similar regulation of growth and differentiation of cells associated with the apical meristem of the root crown in grasses.

Transcription factors are key proteins that control gene activation within the cell; 273 transcription factor DEGs were found in the upregulated and 122 DEGs in the downregulated datasets. Among the highly (log_2_ fold > 2) upregulated transcription factor DEGs were *WRKY*, *AP2* (*APETALA 2*)/ERF (*ETHYLENE RESPONSE FACTORS*), and *NAM*/*NAC* transcription factors, the numbers of which peaked at 2 h post-wounding and after only 1 h of exposure to GLVs. WRKY transcription factors are responsive to wounding and a wide range of other abiotic and biotic stresses [108,109,110], and were previously shown to be induced at high levels (19 DEGs with log_2_ fold changes > 2) after only one hour of exposure to GLVs [42]. In the wound transcriptome, the highly upregulated (log_2_ fold >2) WRKY DEGs were most prevalent at 1 h (6 DEGs) and 2 h (10 DEGs) post-wounding, while the downregulated WRKY DEGs were more prevalent at the later time points. Additionally, upregulated DEGs annotated as GRAS (GAI, RGA, and SCARECROW) and MADS-box containing transcription factors peaked at 1 and 2 h (GRAS) or at 6 h post-wounding (MADS), and were only represented by a single GRAS DEG in the GLV library at the 1 h time point [42]. There were multiple DEGs coding for PHYTOCHROME A SIGNAL TRANSDUCTION 1 (PATI), SCARECROW_LIKE (SCL), and ERF type transcription factors that were upregulated. Interestingly, SCL21- and PATI-type GRAS transcription factors have been shown to interact with ERF transcription factors in the regeneration of excised root tips and to increase the regeneration or callus formation when both factors were overexpressed in *Arabidopsis* [111]. In tissue culture, auxin and cytokinin levels are commonly manipulated to effect callus, root, or shoot formation. Similarly, changes in hormone biosynthesis and response genes have been reported during wound responses in *Arabidopsis* [112]. Among the cytokinin-related upregulated DEGs were several that were identified as LONELY GUYs (LOGs) biosynthetic genes, which were present at the early time points, peaking with six DEGs at the 2 h time point. There were also cytokinin oxidases, glucosyltransferases, and potential cytokinin receptors in the upregulated DEGs, but there were very few cytokinin-related genes in the downregulated DEGs. There were many DEGs identified as auxin conjugate/amino acid hydrolases, auxin-repressed protein-like, auxin-responsive proteins/factors, and auxin-induced proteins in both the upregulated and downregulated DEGs, perhaps important for the redistribution of auxin in response to wounding to adjust the local cytokinin/auxin ratios to promote wound healing and tissue regrowth.

The replacement of lost tissue after wounding requires rapid controlled growth. Some proteins involved in signal transduction networks that result in ROS production have also been shown to participate in cell division [99,113,114]. NADPH oxidases, phospholipase D (PLD), and phosphatidylinositol 3-kinase (PI3K) are also involved in microtubule polymerization and organization [99]. Furthermore, cell division and growth are regulated by cyclin-dependent kinases (CDKs) [115,116] and cyclin proteins [117]. An analysis of the *Lt* wound transcriptome for DEGs coding for regulators of cellular growth and division revealed 15 cyclin-related DEGs with 7 CDKs, 13 NADPH oxidases, 3 PLDs, and 1 PI3K in the upregulated dataset. The chloroplast is another key component driving growth. Our query found that 7.0% (657) of the upregulated DEGs overall and 5.0% (384) of the downregulated DEGs contained the word “chloroplast” within their annotation. As expected, these DEGs were related to a wide array of pathways, JA biosynthesis (lipoxygenases, fatty acid desaturases, and allene oxide synthase), cytochrome biosynthesis, secondary metabolites (terpenoids, geraniol biosynthesis, polyphenol oxidases, and hydroxysteroid dehydrogenases), and redox pathways (thioredoxin, NAD(P)H-quinone oxidoreductase), to mention a few. This is somewhat expected, as the chloroplast transforms light energy to produce a diverse array of compounds used as building blocks for growth and defense responses in plants. In a related category, 62 photosystem-related DEGs were upregulated and only 15 were found to be downregulated. Table 3 shows the highest levels of upregulated DEGs annotated as chloroplast at 64% (421) and photosystem at 88.7% (55) in the 6 h time point. Both the chloroplast and photosystem DEGs decreased at the 12 h time point, but then increased at 24 h post-wounding. Previous studies have shown increased photosynthesis close to the wound site with localized alterations in the source-sink relationship 24 h after wounding [118]. The increases in chloroplast and photosystem DEGs at the 6 and 24 h time points may be due to the plant needing additional resources for repair and regrowth.

In order to gain insight into the processes involved in the replacement of lost vegetative tissue after severe wounding, the abundance of genes encoding proteins involved in cell wall biosynthesis and modification were investigated. The abundance of three cell-wall-associated DEGs (cellulose synthase [119], xyloglucan endotransglycosylase [120,121], and expansin [122]) were investigated. Cellulose synthase is the main enzyme that produces cellulose, which makes up a large portion of the primary and secondary cell walls. Our analysis revealed the same number of cellulose synthase DEGs in the upregulated and downregulated DEG datasets with similar ranges of differential expression (log_2_ fold changes: average for upregulated DEGs, 1.55; average for downregulated DEGs, −1.41). The greatest number of upregulated cellulose synthase DEGs occurred at the 2 and 6 h time points (all with log_2_ fold changes < 2), while the three DEGs at the 24 h time point showed log_2_ fold changes greater than two. The number of xyloglucan endotransglucosylase/hydrolase DEGs, which encode proteins involved in cell wall extension and strengthening, were significantly greater in the upregulated dataset (6 of 19 total with log_2_ fold changes > 2) compared to the downregulated dataset (1 of 2 with log_2_ fold changes < −2). Expansin DEGs, which encode proteins involved in cell wall loosening, were found to be disproportionately represented in the downregulated dataset. The trends for the upregulation and downregulation of these three cell-wall-associated DEGs were similar to what was observed in the *Lt* GLV transcriptome [42]. Glucan is also a major component in the cell walls of grasses [123] and has been proposed to be involved in auxin-induced cell elongation [124]. Endo-(1,3; 1,4)-β-glucanases can hydrolyze glucan, are induced in response to wounding in rice, and are proposed to be important in cell wall loosening and elongation [125]. The DEGs identified as β-glucanases (1,3; 1,4) were more prevalent in the upregulated (60 DEGs) than in the downregulated (18 DEGs) wound datasets. The upregulated glucanase DEGs were highly induced at the 1 h time point (11/17; log_2_ fold changes > 2/total) and increased at 2 h (20/38), and started decreasing at 6 h (11/32), 12 h (14/30), and 24 h (7/13), while the downregulated glucanase DEGs were fewer, but more prevalent at the later time points (Table 3). There were fewer β-glucanase (1,3; 1,4) DEGs in the GLV dataset, with more upregulated and downregulated DEGs at the early time points. These results suggest that β-glucanases (1,3; 1,4) could be important for cell wall modifications in response to wounding in grasses. Overall, there appears to be a complex regulation of growth within the plant, with the potential reduction in growth in some tissues and increase in growth in others. Unfortunately, using the root crown and aerial portions of the plant to generate the transcriptome produced a more global transcriptional profile for the plant, which provides limited information on tissue-specific expression. Therefore, there is a limited amount of information that we can derive about the specific interactions occurring at the tissue level by comparing the ratio and relative abundance of these cell wall modifying enzymes and proteins in the wound database.

The transportation of ions, peptides, small molecules, lipids, and macromolecules across membranes and to their proper location is vital for proper functioning of the cell [126,127,128]. The transcriptome analysis revealed that 6.5% (613) of the total upregulated DEGs and 4.5% (344) of the downregulated DEGs had annotations for transport. The transportation process is essential for cell viability, so it was not surprising to find a relatively even distribution of DEGs across all time points, with the greatest number overall (294) observed at 6 h post-wounding (Table 3), but with a greater number having a higher level of differential expression (log_2_ fold changes > 2) at 2 h post-wounding (Table 3). Interestingly, DEGs identified as calcium-transporting ATPases were found to be upregulated at 1 h (4/8; log_2_ fold changes > 2/total), 2 h (8/8), and 6 h (2/2) time points in the wound dataset, and in the 1 h (4/6) and 2 h (1/5) time points in the GLV dataset [42]. Additionally, there were DEGs for many other types of transporters present in the databases including sugar transporters, which were more prevalent in the upregulated DEGs peaking at 2 to 6 h post-wounding. The ABC transporter DEGs were highly upregulated (68/154; log_2_ fold changes > 2/total) with a peak at 2 h post-wounding (35/80) (Table 2 and Table 3), but some ABC transporter DEGs were also downregulated (62/108) (Table 2) with a peak at 6 h (53 downregulated DEGs) post-wounding. Another important activity for cell viability is that of transferases. Overall, they comprised 6.8% of the total upregulated DEGs and 7.2% of the downregulated DEGs (Table 2). The values for other upregulated and downregulated DEGs related to a wide range of other cellular processes such as synthases, oxidases, reductases, dehydrogenases, and hydrolases are described in Table 2 and Table 3. In analyzing the GO terms, the protein folding subcategory was found to be over-represented by downregulated DEGs over the course of the study. As expected, our analysis of the DEG databases found that stabilizing proteins, such as heat shock/chaperones [129,130], were over-represented in the downregulated DEGs by roughly 3-fold (183) as compared to the upregulated DEGs (63).

In addition to growth, plants respond to wounding by producing defense-related compounds and proteins. In 1972, ground-breaking research conducted by Green and Ryan [1] described for the first time the wound induction of protease inhibitors (PIs) both locally and systemically in plant leaves as a defense mechanism against insects. This discovery was the progenitor for the molecular characterizations of the plant wound responses that we have today. Protease inhibitors (PIs) are an important component of the plant defense response against insects [1,131,132,133]. They inhibit digestive proteinases in the insect gut, which can cause reduced growth and development of the insect [131,132,133]. Wound-induced PIs have been best characterized in the *Solanaceae* plant species [131,132,133]. However, induction of PIs has also been described in cereal grasses, such as the wound-induced systemic accumulation of a transcript encoding a Bowman–Birk trypsin inhibitor related protein in maize seedlings [134], and a maize PI gene produced in response to wounding and a fungal infection [135]. In addition, a maize proteinase inhibitor was induced in response to wounding and insect feeding, and its inhibitory effects on *Spodoptera littoralis* larvae were demonstrated [61]. In their molecular characterization of a proteinase inhibitor in *Brachypodium*, Mur et al. [63] suggested the conservation of some of the defense signaling pathways between dicotyledonous plants and grasses. The analysis of the *Lt* wound library revealed 25 PI DEGs in the upregulated and 8 PI DEGs in the downregulated dataset. These included Bowman–Birk type inhibitors, subtilisin-chymotrypsin inhibitors, cysteine proteinase inhibitors, Kunitz protease inhibitors, fungal protease inhibitors, and others annotated as wound-induced protease inhibitors or just as PIs. These results together with previous studies support the conservation of defense signaling pathways between dicotyledonous plants and grasses [63].

Interestingly, we also found a significant number of upregulated DEGs in categories associated with disease (97), pathogen (44), chitinase (30), β-1,3-glucanase (30), and Avr9 interactive proteins (40) in the upregulated wound dataset (Table 2). Their abundance was fairly evenly spread across the time points, with the highest levels observed in the 2 h time-point dataset (Table 3). This is not surprising since there have been many examples of pathogen-related genes being induced by wounding in other plant species [136,137,138,139,140,141]. Transcriptional profiling analyses performed in *Arabidopsis* revealed a number of wound-responsive genes encoding proteins involved in pathogen responses [4]. These include signaling molecules for the pathogen resistance pathway and enzymes required for cell wall modification and secondary metabolism. In rice, six members of the pathogenesis-related 1 gene family were shown to be induced by wounding, and SA and/or JA activated a number of them [142]. Both chitinases and β-1,3-glucanases have been shown to be induced by wounding and pathogens, and to act synergistically to inhibit the growth of phytopathogenic fungi [137,138,143]. The severe wounding and damage to tissues present a significant breach in the physical barriers that act as a passive defense against pathogens. Therefore, the wound induction of disease-related DEGs is most likely a proactive measure by the plant to defend against opportunistic infections by pathogens due to loss of integrity of the plant’s natural physical barriers used to protect the plant [144]. The relative abundance of pathogen/disease-related DEGs and their presence over the course of 24 h, as shown in Table 3, are consistent with this concept.

### 2.5. Validation of RNA-Seq with qRT-PCR

**Figure 5:**
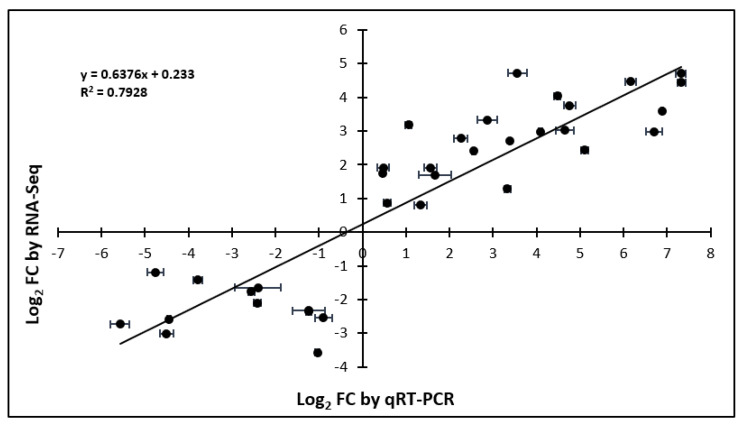
qRT-PCR verification of RNA-Seq results of *Lolium temulentum* in response to wounding. Correlation between the relative quantification between control and wounded samples at different time points (1, 2, 6, 12, and/or 24 h) inferred by RNA-Seq analysis (log_2_ fold changes) and qRT-PCR analysis (log_2_ fold changes with standard error bars). The values represent the log_2_ fold of the relative fold change between the control and the wounded plant at various time points. Regression line and correlation coefficient are shown in the figure.

Twelve DEGs (seven upregulated and five downregulated) were selected for qRT-PCR analysis. The genes were selected to give a range of differential expression across multiple time points. A comparison of the log_2_ fold changes in expression for RNA-Seq and qRT-PCR was graphed and is shown in Figure 5. The trinity numbers and values for the log_2_ fold changes in expression for RNA-Seq and qRT-PCR are provided in Table S5. The results showed general agreement between the RNA-Seq and the qRT-PCR results for the upregulation or downregulation of selected genes in response to wounding.

## 3. Materials and Methods

### 3.1. Plant Materials

*Lolium temulentum* L. (*Lt*, Darnel ryegrass) cv. Ceres seeds were planted, five seeds per pot, in TSD4 square pots (8.8 cm × 8.8 cm × 10 cm, 540 mL volume; McConkey Co., Sumner, WA) with Sun Gro Professional MM840 PC RSi (Sun Gro Horticulture, Hubbard, OR). *Lolium* plants were grown in Conviron PGR14 or PGR15 growth chambers (Conviron, Winnipeg, Canada), under 14 h photoperiods at 23 °C day and 18 °C night temperatures and fertilized with Technigro 20-18-20 all-purpose fertilizer (Sun Gro Horticulture, Hubbard, OR) weekly. All experiments were conducted using seeds from increases of *Lolium temulentum* cv. Ceres seeds originally provided by Dr. Lloyd T. Evans (CSIRO, Canberra Australia) in 2001.

### 3.2. Plant Treatments

Plants were wounded by pinching off sections of tillers using a pair of pliers. Tillers were pinched off three to four times beginning at the top of the tiller and continuing down the leaf blade to between three and nine cm above the root crown; the removed tissue was discarded. Wounding occurred 1.5 h after the lights were turned on, and all time points were collected during the 14 h day-length cycle. Five plants were collected at each time point: 1, 2, 6, 12, and 24 h post-wounding. Three independent biological replicates were collected. All replicate plants were grown in the same growth chamber. Approximately 16 h before conducting the experiment, treated (wounded) and control (unwounded) plants were placed into two separate growth chambers. At the designated time points, the aerial portions of the plant and root crown were collected, placed in foil packets, quickly submerged in liquid nitrogen, and stored at −80 °C. Control samples were collected at the same time as treatment samples. During the collection of control samples, the upper leaf material of the tiller was removed and discarded so that the same portion of the plant would be analyzed for both the wound treated and control tissues.

A separate and independent wound experiment (treatment and control plants) was performed to generate samples for qRT-PCR verification of RNA-Seq results. The plants were grown and treated as described above, with one biological replicate for each time point consisting of pooled tissues from five individually treated plants. Control and wounded samples were collected at 1, 2, 6, 12, and 24 h post-wounding.

### 3.3. RNA Sample Preparation and Illumina Sequencing

Thirty library preparations from three biological replicates of two treatments (control and wound) at five time points, as described above, were prepared. For qRT-PCR experiments, RNA was extracted from one biological replicate (5 pooled plants) of two treatments (control and wounded) at five time points. Total RNA was extracted from plant tissues using Trizol (Invitrogen, Carlsbad, CA), according to the manufacturer’s instructions. DeNovix DS-11 spectrophotometer (DeNovix Inc., Wilmington, DE) was used to measure RNA concentration and quality. RNA was assessed for quality, processed, and submitted for sequencing as described in [42], except the final step of DNase deactivation after DNase treatment was not performed. Instead, the RNA Clean and Concentrate Kit (Zymo Research, Irvine, CA) was used to purify the samples according to the manufacturer’s instructions. The RNA samples were then prepared using the Wafergen RNA kit and sequenced on the Illumina HiSeq 3000 using a 100 bp paired-end run.

### 3.4. Transcriptome Assembly and Analysis

Raw sequences were quality- and adapter-trimmed with Cutadapt (-q 15, 10) [145]. Alignments were done with BWA-MEM [146] against the *Lolium* transcriptome [42]. SAMtools [147] was used for downstream processing of the alignments. Cuffdiff [148] was used to calculate reads per transcript and to identify differentially expressed genes. Cummerbund [149] was used for visualization of differential expression. Genes were annotated by alignment against grass proteins in the UniProt TrEMBL database [150] using NCBI BLASTx [151]. A reference set of GO identifiers [152,153,154] was created for the full *Lolium* transcriptome [42] using the UniProt database. WEGO 2.0 [155] was used to identify GO category differences between the reference transcriptome and upregulated and downregulated DEGs. Significant differences in GO classifications were calculated by a Chi-Square test for each GO term. UpSet bar charts were constructed using UpSetR [156] in R (R Core Team, 2017).

### 3.5. Validation of RNA-Seq with qRT-PCR

The validation of RNA-Seq results was conducted by comparing differential expression values of five downregulated DEGs and seven upregulated DEGs to those obtained using qRT-PCR. An independent experiment was performed to obtain samples for qRT-PCR. Primer3Web (v. 4.1.0) was used to design primers. The trinity number, primer sequences, amplicon length, and primer efficiencies are listed in Table S5. Primer evaluation, cDNA preparations, reaction mixture and conditions (but with an annealing temperature of 58 °C), and qRT-PCR data analysis were performed as previously described [42]. Eukaryotic elongation factor 1−α (eEF1–α) and ubiquitin 5 (UBQ5) [157] were used as reference genes for sample expression normalization. For all genes except one, no template control NTC samples had Cq values above 34 and most were undetected. One primer pair had an average NTC Cq of 33, which was 6 Cqs above the latest sample Cq of 27 for that gene.

## 4. Conclusions

**Figure 6:**
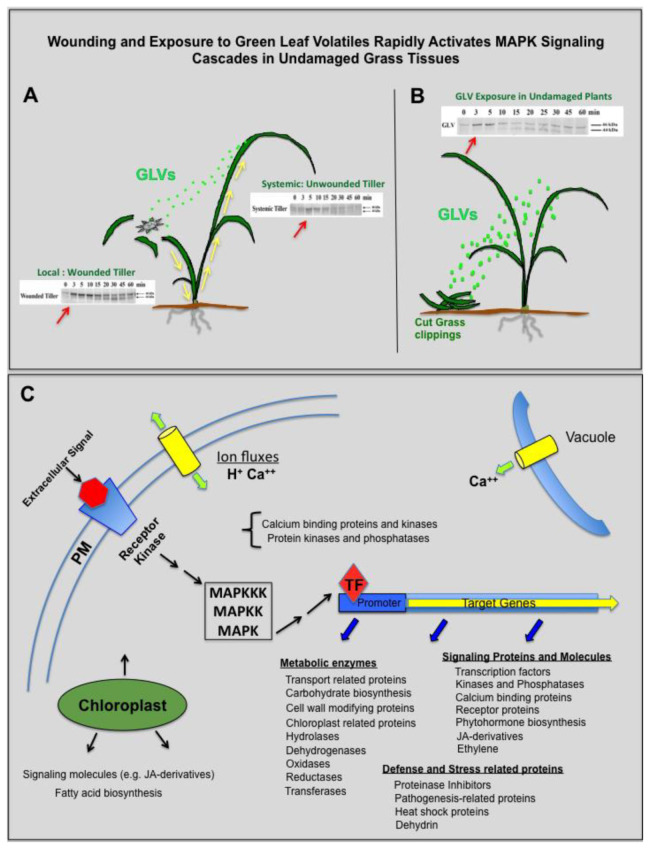
Wounding and exposure to green leaf volatiles (GLVs, green dots) rapidly activate mitogen-activated protein kinase (MAPK) signaling cascades in undamaged grass tissues. (**A**) Mechanical wounding rapidly activates both a 46 kDa MAPK and a 44 kDa MAPK (red arrows) locally and systemically in the unwounded tillers [66] using intra-plant signals (yellow arrows) or GLV chemical signals. (**B**) GLV also rapidly activates these MAPKs in nearby undamaged plants after just one minute of exposure [67,68]. (**C**) Diagram of proposed molecular events occurring within the cell after mechanical wounding. Abbreviations: Ca, calcium; GLVs, green leaf volatiles; JA, jasmonic acid; MAPK, mitogen-activated protein kinase; TF, transcription factors.

The analysis of the *Lt* wound transcriptome revealed the upregulation of genes encoding a wide array of proteins involved in signaling, transport, defense, and metabolic processes. In response to wounding, grasses produce signals that rapidly activate MAPK, not only in the damaged tiller, but also in adjacent tillers within three minutes of wounding (Figure 6A) [66]. These MAPK signaling proteins could be activated by intra-plant signals such as reactive oxygen species, hydraulic, electrical, and/or phytohormone based signals or by wound-released airborne GLV chemical signals. Intra-plant-based signaling molecules would have to travel down the pseudostem of the wounded tiller through the dense root crown and back up into the adjacent unwounded tillers to generate a response in the adjacent tiller (Figure 6A, yellow arrows). Interestingly, GLV released from grass clippings are also able to activate rapidly the MAPK in unwounded neighboring plants (Figure 6B) [67,68]. Not surprisingly, genes encoding MAPK and receptor kinases were found to be upregulated in the wound transcriptome (Figure 6C). In our proposed wound response pathway (Figure 6C), extracellular signals (phytohormones or biophysical- or chemical-based) released from damaged plant tissues after wounding interact with plasma membrane-based receptor kinases. These interactions, combined with ion fluxes within and out of the cell, lead to the transmission of the signals via effector proteins (e.g., MAPK signaling cascades and transcription factors), resulting in the activation and transcription of genes coding for an array of proteins involved in diverse cellular and molecular functions as shown in Figure 6C. Included in this protein array are more signaling proteins and molecules, defense- and stress-related proteins, and metabolic enzymes. These include different families of transcription factors, kinases/phosphatases, and proteins involved in the synthesis and perception of phytohormones. In addition to signaling DEGs, a significant portion of the DEGs were related to transport of cellular components such as ions, peptides, small molecules, lipids, and macromolecules, and to proteins associated with chloroplast function and transferase activity, which reflects the dynamic processes involved in the wound stress response. Mechanical wounding also induced genes coding for defense-related proteins, such as proteinase inhibitors, and a variety of pathogenesis-related DEGs, such as chitinases and β-1,3-glucanases. The analysis of this wound transcriptome is the first step in identifying the type of defense compounds produced, and the molecular components and pathways used by forage and turf grasses to respond to wounding. The information gained from the analysis will provide a valuable molecular resource that will be used to develop approaches that can improve the recovery, regrowth, and long-term fitness of forage and turf grasses before and after cutting or grazing.

## Figures and Tables

**Table 1:** Sequence reads (forward and reverse) and percent alignments for wound and control plant libraries.

Time (h)	Treatment	Replicate 1		Replicate 2		Replicate 3	
		Reads	Alignment	Reads	Alignment	Reads	Alignment
**1**	**Control**	59843094	98.5%	58101312	98.2%	59686650	98.0%
**2**	**Control**	64916262	98.5%	74641320	98.0%	67418496	98.0%
**6**	**Control**	64740740	98.6%	68759474	98.0%	71426244	97.8%
**12**	**Control**	48624754	98.4%	43314674	98.3%	37368582	98.4%
**24**	**Control**	64365702	98.6%	68469240	98.2%	68572898	97.9%
**1**	**Wound**	62072772	98.6%	62531754	98.0%	52132476	98.1%
**2**	**Wound**	69339124	98.3%	83197276	97.9%	57943888	98.2%
**6**	**Wound**	62301400	98.5%	61347708	98.0%	61003562	98.1%
**12**	**Wound**	55820584	98.2%	47114420	98.2%	60013782	98.3%
**24**	**Wound**	66900286	98.4%	72082570	98.0%	59419184	98.1%

**Table 2:** Biological functional analysis of DEG database.

	UP	DOWN			
**Total DEGs (All)**	9413	7704			
**Unannotated Sequences**	1959	1956			
	**DEGs**			**DEGs**	
**Keyword Search**	UP () *	DOWN () *	**Keyword Search**	UP () *	DOWN () *
Kinase	637 (280)	348 (143)	Cytochrome	173 (89)	131 (52)
Receptor kinase	281 (66)	112 (26)	Cytochrome P450	133 (69)	68 (33)
Mitogen-activated protein kinase/MAPK	18 (4)	6 (1)	Dehydrogenase	259 (121)	211 (79)
Phosphatase	169 (70)	76 (34)	Calcium/calmodulin	130 (40)	54 (24)
Receptor	431 (229)	236 (113)	Auxin	76 (33)	45 (12)
LRR receptor	46 (23)	15 (5)	Cytokinin	20 (9)	2 (0)
Systemin/brassinosteroid receptor	16 (3)	3 (1)	Salicylate/salicylic	22 (10)	4 (1)
Transcription	382 (194)	218 (81)	Ethylene	78 (31)	31 (13)
Transcription factor	273 (122)	122 (41)	Abscisic acid	18 (4)	6 (0)
WRKY	19 (14)	9 (6)	Gibberellin	20 (12)	8 (3)
BZIP	27 (9)	8 (3)	Lipoxygenase	24 (13)	12 (3)
Heat shock/chaperone	63 (29)	183 (38)	Jasmonate/jasmonic	24 (12)	9 (3)
Protease/proteinase/peptidase	264 (112)	204 (73)	Lipase	84 (29)	46 (13)
Protease/proteinase/peptidase Inhibitor	25 (7)	8 (0)	Phospholipase	34 (8)	17 (4)
Ubiquitin	124 (42)	79 (29)	Proline	74 (31)	50 (15)
Chloroplast/chloroplastic	657 (154)	384 (80)	Glycine betaine/proline transporter	5 (2)	1 (1)
Photosystem	62 (10)	15 (8)	Phenylalanine ammonia-lyase	15 (8)	1 (1)
Chlorophyll	44 (13)	27 (9)	Glucanase	60 (27)	18 (7)
Phytochrome	27 (10)	7 (3)	Ferric reductase	8 (0)	9 (0)
Sucrose	54 (21)	25 (5)	Expansin	9 (4)	16 (4)
Glucose	111 (47)	94 (19)	Xyloglucan endotransglycosylase	19 (6)	2 (1)
Glucan	106 (50)	49 (17)	Cellulose synthase	19 (3)	19 (2)
Glucosyltransferase	107 (46)	43 (16)	ATP	433 (158)	377 (117)
Monosaccharide transporter	16 (9)	2 (0)	ATPase	116 (49)	95 (31)
Amino acid	95 (43)	45 (15)	GTP	57 (18)	72 (16)
Amino acid transporter	70 (29)	39 (13)	GTPase	21 (10)	38 (10)
Membrane	379 (150)	285 (117)	Dehydrin	5 (2)	0
Channel	64 (23)	31 (14)	Thioredoxin	27 (5)	14 (3)
ABC transporter	154 (68)	108 (62)	Thaumatin	11 (9)	6 (1)
Transport	613 (253)	344 (150)	Inhibitor	80 (34)	39 (7)
Transferase	641 (322)	557 (202)	Disease	97 (44)	41 (20)
Esterase	130 (63)	89 (31)	Pathogen	44 (18)	22 (7)
Invertase	40 (15)	21 (4)	Chitinase	30 (21)	11 (3)
Synthase	404 (192)	315 (126)	Avr9	40 (21)	7 (2)
Synthetase	153 (88)	144 (74)	Avr9 Cf-9	34 (19)	6 (2)
Reductase	272 (119)	225 (84)	Allene oxidase	6 (3)	0
Oxidase	238 (100)	133 (42)	Allene cyclase	1 (0)	1 (1)
Oxygenase	181 (89)	71 (31)	12-Oxophytodienoic		
Peroxidase	57 (26)	32 (7)	acid reducase	8 (4)	1 (0)
Hydrolase	319 (141)	152 (69)	ACC oxidase	29 (17)	5 (1)

* All DEGs based on values with a false discovery rate < 0.05 and *p*-value < 0.01; () DEGs with |log_2_ fold changes| > 2.

**Table 3:** Abundance of DEG functional class per time point.

**Total UP DEG**	**1 Hour**	**2 Hour**	**6 Hour**	**12 Hour**	**24 Hour**
	1382	3484	4067	3418	2421
**DEG Designation**	All UP () *	All UP () *	All UP () *	All UP () *	All UP () *
Kinase	140 (81)	321 (135)	272 (90)	177 (26)	125 (40)
Phosphatase	34 (22)	82 (39)	83 (31)	52 (11)	57 (20)
Calcium/calmodulin	46 (16)	89 (22)	34 (11)	20 (2)	18 (9)
Transcription factor	67 (39)	151 (72)	125 (46)	68 (11)	63 (13)
Transcription	85 (52)	187 (94)	175 (67)	93 (19)	95 (31)
Synthase	74 (39)	158 (86)	212 (86)	105 (29)	122 (47)
Oxidase	34 (21)	99 (39)	116 (49)	74 (19)	75 (31)
Reductase	34 (23)	89 (48)	144 (53)	60 (11)	105 (28)
Peroxidase	8 (7)	22 (9)	33 (16)	24 (12)	15 (6)
Dehydrogenase	42 (25)	106 (56)	149 (62)	66 (7)	81 (20)
Transport	112 (66)	280 (138)	294 (98)	214 (38)	189 (60)
ABC transporter	21 (9)	80 (35)	43 (20)	41 (5)	35 (14)
Monosaccharide transporter	5 (2)	10 (10)	13 (8)	10 (6)	4 (1)
Chloroplast	75 (49)	141 (73)	421 (72)	154 (11)	307 (32)
Photosystem	2 (1)	2 (1)	55 (6)	4 (2)	35 (1)
Disease	22 (5)	49 (28)	35 (11)	34 (8)	28 (6)
Pathogen	11 (4)	31 (15)	20 (3)	21 (9)	13 (4)
Chitinase	6 (4)	22 (18)	20 (12)	13 (8)	4 (1)
Glucanase	17 (11)	38 (20)	32 (11)	30 (14)	13 (7)
Allene oxide synthase	5 (2)	5 (3)	3 (1)	1 (0)	0
ACC oxidase	5 (2)	9 (6)	12 (6)	8 (3)	13 (8)
Thioredoxin	3 (2)	5 (4)	17 (5)	7 (0)	11 (1)
Xyloglucan endotransglycoslase	1 (0)	10 (3)	11 (5)	7 (2)	5 (3)

* All DEGs based on values with a false discovery rate < 0.05 and *p*-value < 0.01; () DEGs with |log_2_ fold changes| > 2.

## Supplementary Materials

The following are available online at https://www.mdpi.com/2223-7747/9/6/780/s1, Table S1: *Lolium* transcriptome information, Table S2: List of upregulated and downregulated DEGs between wounded and control plants at different time points, Table S3: Table of unique and shared DEGs, Table S4: Data for UpsetR plots shown in Figure 4 and all upregulated vs all downregulated DEG Venn, Table S5: Values for RNA-Seq vs qRT-PCR comparisons and qRT-PCR primer information.
